# Preservation of Mechanical and Morphological Properties of Porcine Cardiac Outflow Vessels after Decellularization and Wet Storage

**DOI:** 10.3390/biomimetics8030315

**Published:** 2023-07-17

**Authors:** David Sergeevichev, Maria Vasiliyeva, Elena Kuznetsova, Boris Chelobanov

**Affiliations:** 1 NMRC Named after Academician E.N. Meshalkin of the Ministry of Health of the Russian Federation, Novosibirsk 630055, Russia; 2 Vorozhtsov Institute of Organic Chemistry SB RAS, Novosibirsk 630090, Russia; 3 V. Zelman‘s Institute of Medicine and Psychology, Novosibirsk State University, Novosibirsk 630090, Russia; 4 Institute of Chemical Biology and Fundamental Medicine SB RAS, Novosibirsk 630090, Russia

**Keywords:** bioprosthetic valve, decellularization, tissue storage, uniaxial mechanical testing, tissue engineering, extracellular matrix

## Abstract

Widely used storage methods, including freezing or chemical modification, preserve the sterility of biological tissues but degrade the mechanical properties of materials used to make heart valve prostheses. Therefore, wet storage remains the most optimal option for biomaterials. Three biocidal solutions (an antibiotic mixture, an octanediol-phenoxyethanol complex solution, and a glycerol-ethanol mixture) were studied for the storage of native and decellularized porcine aorta and pulmonary trunk. Subsequent mechanical testing and microstructural analysis showed a slight increase in the tensile strength of native and decellularized aorta in the longitudinal direction. Pulmonary trunk elongation increased 1.3–1.6 times in the longitudinal direction after decellularization only. The microstructures of the tested specimens showed no differences before and after wet storage. Thus, two months of wet storage of native and decellularized porcine aorta and pulmonary trunks does not significantly affect the strength and elastic properties of the material. The wet storage protocol using alcohol solutions of glycerol or octanediol-phenoxyethanol mixture may be intended for further fabrication of extracellular matrix for tissue-engineered biological heart valve prostheses.

## 1. Introduction

Prosthetic valve replacement is the gold standard for treating severe heart valve disease. However, even the most advanced prosthetic valves have drawbacks. Mechanical valves are durable but require strict adherence to lifelong anticoagulant therapy. In addition, their functioning is accompanied by some acoustic effects, which are annoying and significantly reduce the patient’s quality of life [1,2].

The low risk of thromboembolism with bioprosthetic valves is offset by a limited lifetime due to progressive material degeneration and the development of complications associated with surface mineralization. The use of donor tissue to replace defective heart valves at any age remains the best-choice option [1,3,4]. Excellent hemodynamic properties and resistance to potential infection and thrombosis distinguish allografts from xenogeneic conduits and artificial valves [5,6]. To date, only allografts ensure a minimal probability of the development of structural defects of the prosthetic material and the associated risk of repeated interventions [1,6,7,8]. However, the deficiency of donor material keeps the development of new tissue-engineered valve prostheses highly relevant.

Naturally occurring connective tissue scaffolds (native biological materials and decellularized tissues) for cardiac tissue engineering appear to be preferable to constructs made from chemically modified biological materials (e.g., glutaraldehyde or diepoxy compounds) or degradable biopolymers [6,9]. The decellularization of biological materials preserves the mechanical anisotropy of the extracellular matrix of the native valve or vascular tissue and significantly reduces the risk of immune-mediated bioprosthesis degeneration after implantation [10,11,12]. These materials are admirably integrated into the modern concept of tissue engineering in situ because of their ability to be repopulated with recipient cells with high efficiency after implantation [6]. Excluding the common variants of irreversible aggressive chemical treatment of biological tissues with glutaraldehyde or epoxide, which irreversibly changes the connective tissue framework (CTF), two methods of preimplantation tissue treatment remain—cryofreezing and wet storage [7,13]. Each of them has its own disadvantages, so there is no single approach to choosing one or the other method for the long-term preservation of prosthetic material.

The main advantage of cryopreservation and the existence of a tissue bank is the on-call availability of cardiac surgery centers. Harvesting and cryopreserving heart valves require specialized equipment and trained personnel [14]. Therefore, the influence of the human factor and the possibility that the freeze/thaw technology may be compromised, resulting in the disruption of the morphologic structure of the CTF, should be considered. In this case, macroscopic damage can be detected in preparation for implantation, and the valve can be discarded. Microscopic damage, however, will only manifest itself in the long term as a functional failure of the prosthesis due to mineralization or structural degradation and degeneration [15,16,17].

The method of wet storage of materials in a solution of several antibiotics without cryopreservation avoids this type of damage. It helps to reduce the frequency of reoperations associated with early damage to the prosthesis [8]. However, wet storage of allografts is only possible for a short period of time, up to 2 weeks [18,19]. It is also known that residual amounts of antimicrobial drugs in the allograft tissue can cause the development of non-specific complications (allergic reactions, inflammation, and delayed bacteremia during contamination of the prosthesis) [20]. Surprisingly, the role of glycerol solutions in biomaterial storage has been underappreciated. Because of their ability to preserve the structure of extracellular matrix proteins, they are applied in the manufacture of skin and ligament grafts and may potentially be used for the storage of native and decellularized heart valves and peripheral vessels [21,22]. In addition to the use of classical methods of wet storage, new agents with biocidal properties but without pronounced toxic effects on biological organisms are being sought. Thus, octanediol, phenoxyethanol, and other preservatives used in cosmetology, pharmacy, and pathomorphology are beginning to be used as biocidal agents despite the lack of scientifically validated data on their efficacy and safety [23,24,25].

Previously, we developed several biocidal solutions for the long-term wet storage of connective tissue materials [26,27]. In the present study, we evaluated the effect of decellularization and subsequent 2-month storage in biocidal solutions of different compositions on the mechanical and ultrastructural properties of porcine aortic and pulmonary trunk tissues intended for the further production of tissue-engineered biological prostheses of heart valves.

## 2. Materials and Methods

### 2.1. Sample Processing

Swine aortas (AO) and pulmonary trunks (PT) were collected at a local slaughterhouse and immediately transported to the laboratory in a chilled isotonic solution for further processing. The following manipulations were carried out in a class IIA clean room (Lamsystems, Miass, Russia). After the removal of surrounding tissue residues, the samples were fragmented into 15 × 40 mm strips longitudinally or transversely to the blood flow. Half of the obtained fragments were immediately placed in biocidal solutions, while the other part was subjected to detergent decellularization on the basis of the previously developed protocol [28]. Briefly, a decellularization solution containing 0.5% sodium dodecyl sulfate and 0.5% sodium deoxycholate (both Dia-M, Moscow, Russia) in phosphate salt buffer pH = 7.4 (Biolot, St. Petersburg, Russia) was sterilized by filtration through a 0.22 μm Millex GS syringe filter (Merck KGaA, Darmstadt, Germany) and poured into 200 mL sterile half-liter conical glass flasks containing the tested samples. The weight of the samples in each flask did not exceed 20 g. Decellularization for 24 h and eight subsequent washes for 12 h were performed in a Unimax orbital thermoshaker (Heidolf, Schwabach, Germany) at room temperature and 120 rpm. Sterile phosphate buffer pH = 7.4 (Biolot, Russia) was used as the washing solution.

### 2.2. Biocide Solution Preparation and Sample Storage

Broad-spectrum antibiotic solution (AS): 0.5 g cefazolin (Sintez, Kurgan, Russia), 1 g ampicillin (Sintez, Russia), 100 mg gentamicin (Sintez, Russia), 25 mg metronidazole (Unique Pharmaceuticals, Mumbai, India), 10 mg fluconasol (Sintez, Russia), and up to 500 mL 0.9% sodium chloride solution (Biolot, Russia).

Complex solution (CS): a mixture of phenoxyethanol, 1,2-octandiol, sorbic acid, and ethanol (patent RU 2580621C1) [27].

Glycerol-ethanol solution (GES): glycerin (20%), ethanol (10%), and up to 500 mL 0.9% sodium chloride solution (Biolot, Russia).

The required amounts of solutions were prepared immediately before use, sterilized by filtration through 0.22 μm Millex GS syringe filters (Merck, Germany), and poured into 300 mL sterile half-liter glass conical flasks containing the test tissue samples. Native and decellularized samples (*n* = 10 in each subgroup) were randomly distributed among the four experimental groups and stored at +4 °C for 2 months in a 10-fold excess of the biocide solution (Table 1). Control samples were tested immediately after processing.

### 2.3. Mechanical Testing

AO and PT specimens were prepared as a dogbone shape with working areas of 9 mm (width) × 38 mm (length) using a special cutting die. Tissue thickness (*h*, mm) was measured using an Absolute 227 (Mitutoyo, Sakado, Japan) digital thickness gauge at three points of the sample working area. Each sample was washed in 200 mL of 0.9% sodium chloride solution for 20 min on a shaker at room temperature until assayed.

Uniaxial tensile testing was performed at a constant extension rate of 50 mm/min using an ESM 301L tester (Mark-10 Corporation, Copiague, NY, USA) equipped with a computer-linked force gauge (0–25 N). All samples were stretched until failure. Their strength was evaluated by their ultimate tensile stress (*σ*, MPa) (Equation (1)),

*σ* = *F*/(*h* × *w*) (1)

where *F* is the peak load prior to failure (N), *h* is the mean sample thickness (mm), and *w* is the width (9 mm) of the sample.

Failure strain (*ε*, %) was calculated using Equation (2),

*ε* = ∆*L*/*L* × 100 (2)

where Δ*L* is maximal deformation (mm) and *L* is the initial sample length (mm) between the grips.

Stiffness was evaluated by Young’s modulus (*E*, MPa) (Equation (3)), which was calculated for each specimen at the point with coordinates of “strain below 0.1 MPa” (“low-load modulus”).

*Ε* = *σ*/*ε* (3)

### 2.4. Morphological Study

The histological processing of specimens and preparation of paraffin blocks were performed in accordance with generally accepted methods. Five-micrometer histological sections were prepared on an HM 340E rotary microtome (Thermo Fisher Scientific, Waltham, MA, USA) and dried on Superforst Plus glass slides (Menzel, Braunschweig, Germany). Samples were stained with hematoxylin and eosin (BioVitrum, St. Petersburg, Russia) according to the manufacturer’s recommendations. Microscopic examinations were performed using an AxioSkop 40FL (Carl Zeiss AG, Jena, Germany) with an ADF PRO 08 digital camera and ADF ImageCapture software (all ADF Optics Co., Ltd., Hangzhou, China).

### 2.5. Scanning Electron Microscopy

Samples were dehydrated in increasing concentrations of ethanol (50 to 100%) and then transferred to mixtures of ethanol and hexamethyldisilazane (Sigma-Aldrich, Burlington, MA, USA) in a 1:1 ratio and then to 100% hexamethyldisilazane. Materials were fixed with double-sided carbon tape on a sample stand, sputter-coated with 10 nm gold/palladium, and analyzed with an EVO 10 scanning electron microscope (Carl Zeiss AG, Germany) at an accelerating voltage of 10 kV.

### 2.6. Statistical Analysis

Quantitative data were processed using Statistica 13.0 (TIBCO Software Inc., Palo Alto, CA, USA). The normality of the datasets in each experiment was tested using the Shapiro–Wilk test. Data were reported as median (Me) and interquartile range (Q1:Q3). The Mann–Whitney U test was used to compare two groups, and the Kruskal–Wallis nonparametric analysis of variance with Dunn’s correction was used to compare three or more groups. Statistical significance was set at *p* < 0.05.

## 3. Results

### 3.1. Mechanical Properties

**Figure 1:**
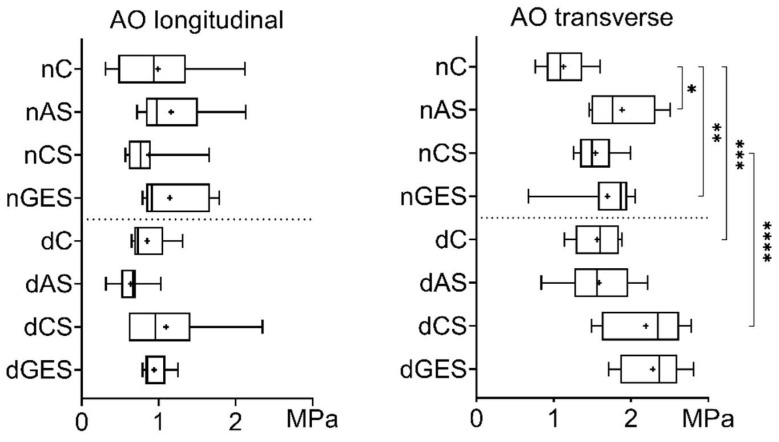
Tensile stress values for aorta samples (Me (Q1:Q3); mean marked as ‘+’). * *p* = 0.005, ** *p* = 0.0196, *** *p* = 0.0293, **** *p* = 0.027.

The use of wet storage solutions did not worsen the strength of native and decellularized aortic (AO) specimens when loaded in the longitudinal direction. There was a trend toward increased strength in the nAS subgroup (1.1617 ± 0.5119 MPa) and in the nGES subgroup (1.1464 ± 0.4405 MPa) compared to the control nC subgroup (0.989 ± 0.5684 MPa) (Figure 1). Among the decellularized specimens, the dCS subgroup has the greatest increase in longitudinal strength compared to the dC subgroup (1.0967 ± 0.6459 MPa vs. 0.8514 ± 0.2441 MPa).

Significant differences were found in the increase in mean transverse tensile strength of native AO specimens. The nAS subgroup showed a 67% increase in strength to 1.88 ± 0.43 MPa (*p* = 0.005), and the nGES subgroup showed a 50% increase to 1.26 ± 0.43 MPa (*p* = 0.0196) compared to the nC control group (1.13 ± 0.43 MPa).

When comparing the decellularized AO specimens, there was also a significant increase in mean tensile strength in the transverse direction. The dCS subgroup showed a 40% increase in mean strength to 2.19 ± 0.49 MPa, and the dGES subgroup showed a 46% increase to 2.28 ± 0.42 MPa compared to the dC control group (1.56 ± 0.29 MPa). Interestingly, after decellularization, the mean transverse tensile stress of the AO specimens increased by 38% to 1.56 ± 0.29 MPa (*p* = 0.0293), while their longitudinal stress decreased slightly.

**Figure 2:**
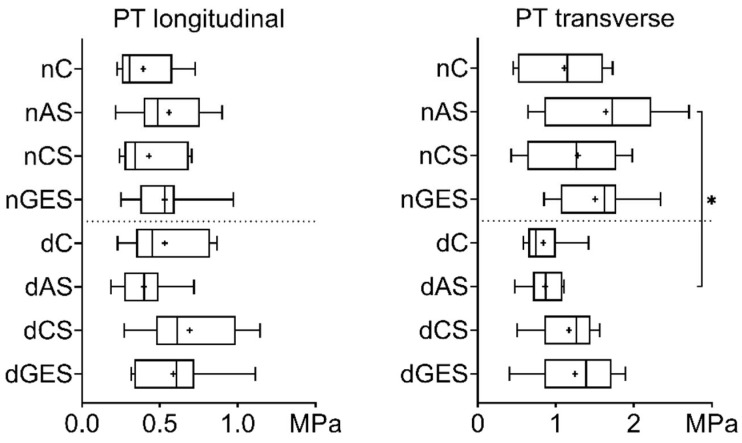
Tensile stress values for pulmonary trunk samples (Me (Q1:Q3); mean marked as ‘+’). * *p* = 0.035.

A strength analysis of the pulmonary trunk (PT) specimens showed that 60 days of wet storage of the biomaterials did not degrade the mechanical properties. The greatest increase in the longitudinal strength of native PT specimens compared to control (0.393 ± 0.187 MPa) was achieved in the nAS (0.560 ± 0.219 MPa) and nGES (0.531 ± 0.227 MPa) subgroups (Figure 2). Among the decellularization subgroups, the largest increase was seen for dCS vs. dC (0.691 ± 0.295 MPa vs. 0.532 ± 0.252 MPa). No significant differences were observed.

An increase in the transverse tensile strength of native PT was achieved in the nAS (1.641 ± 0.721 MPa) and nGES (1.506 ± 0.517 MPa) subgroups compared to the control nC group (1.110 ± 0.508 MPa). Similarly, decellularized PT samples in the dGES (1.247 ± 0.522 MPa) and dCS (1.170 ± 0.362 MPa) subgroups showed increased tensile strength compared to dC (0.840 ± 0.277 MPa). However, when comparing the whole PT group before and after treatment, a decrease in strength in the transverse direction is evident after decellularization (nAS vs. dAS: 1.641 ± 0.721 MPa vs. 0.840 ± 0.277 MPa, *p* = 0.035), but the strength in the longitudinal direction is preserved.

**Figure 3:**
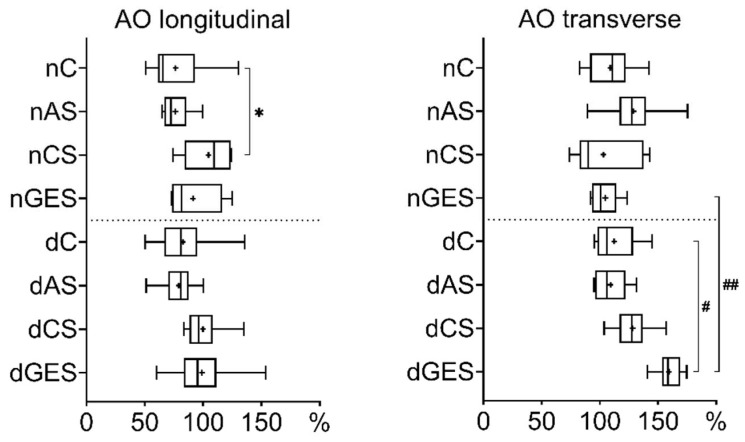
Relative elongation values for aorta samples (Me (Q1:Q3); mean marked as ‘+’). * *p* = 0.0277, # *p* = 0.0038, ## *p* = 0.0012.

The relative elongation of native and decellularized AO specimens in the longitudinal direction did not deteriorate after wet storage in all types of solutions compared to the control (Figure 3). The largest change was found in the nCS subgroup (nC vs. nCS: 76.41 ± 24.60% vs. 104.65 ± 20.37%, *p* = 0.0277).

The transverse elongation study showed an increase in the dGES subgroup (159.53 ± 11.30%) compared to the control dC subgroup (112.29 ± 18.71%, *p* = 0.0038) and the native nGES material (104.7 ± 12.29%, *p* = 0.0012).

**Figure 4:**
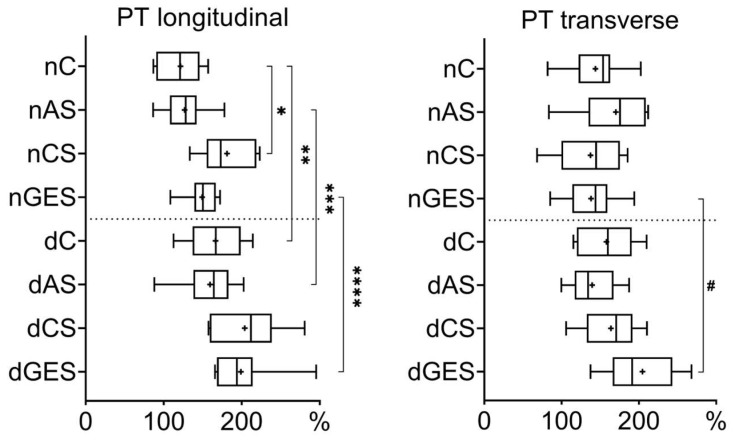
Relative elongation values for pulmonary trunk samples (Me (Q1:Q3); mean marked as ‘+’). * *p* = 0.0057, ** *p* = 0.0256, *** *p* = 0.0115, **** *p* = 0.0033, # *p* = 0.0262.

An analysis of the relative elongation of the PT materials showed a significant increase in values during longitudinal tensile strain. In the nCS subgroup (181.23 ± 32.63%), the relative longitudinal elongation value exceeded the control nC values (121.60 ± 27.75%) by 1.5 times (*p* = 0.0057) (Figure 4).

Decellularization also resulted in an increase in the longitudinal elongation of PT materials. The dC subgroup (166.55 ± 36.10%) had a 1.37-fold increase (*p* = 0.0256) compared to nC, the dAS subgroup (159.41 ± 33.46%) had a 1.26-fold increase (*p* = 0.0115) compared to nAS (126.90 ± 25.89%), and the dGES subgroup (199.00 ± 41.77%) had a 1.33-fold increase (*p* = 0.0033) compared to nGES (149.23 ± 22.07%).

The transverse elongation of PT materials within the native and decellularized subgroups was not significantly different. A 1.5-fold (*p* = 0.0262) increase in elongation was greatest in the dGES subgroup (204.67 ± 46.67%) compared to the untreated material in the nGES subgroup (138.10 ± 35.12%).

**Figure 5:**
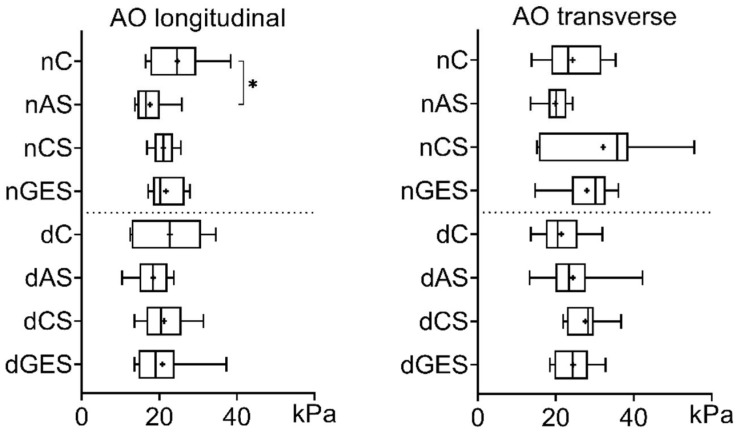
Young’s modulus values for aorta samples (Me (Q1:Q3); mean marked as ‘+’). * *p* = 0.043.

The Young’s modulus of native AO specimens measured in the longitudinal direction decreased after storage in antibiotic solution (nC vs. nAS: 24.64 ± 6.93 kPa vs. 17.56 ± 4.37 kPa, *p* = 0.043). In other cases, there were no significant differences between native and decellularized samples, regardless of the direction of stretching within either group or when compared to corresponding control samples (Figure 5).

**Figure 6:**
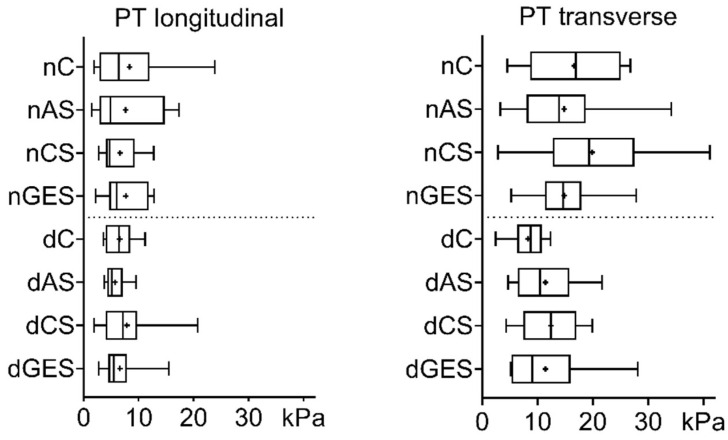
Values of the Young’s modulus for the pulmonary trunk specimens (Me (Q1:Q3); mean marked as ‘+’).

Decellularization and prolonged wet storage had no significant effect on the Young’s modulus of the pulmonary trunk specimens. The overall slight decrease in this index is noticeable when comparing the native and decellularized groups (Figure 6).

### 3.2. Microstructural Analysis

In the control aortic specimens (nC group), the relative arrangement and regular crimp (waviness) of the elastin fibers can be clearly seen. It is important to note that the thickness of the elastin fibers themselves is approximately the same within the sections. After decellularization, this tortuosity is somewhat reduced, but it is important to maintain the uniformity of the thickness of the elastin fibers (dC droup). A similar regularity in the mutual arrangement of different elements of the CTF can be observed in the corresponding scans.

At the end of the storage of the samples in the experimental solutions, the most pronounced undesirable changes were detected in the dAS group, where uneven thinning of elastin fibers was found in combination with the swelling and homogenization of collagen.

**Figure 7:**
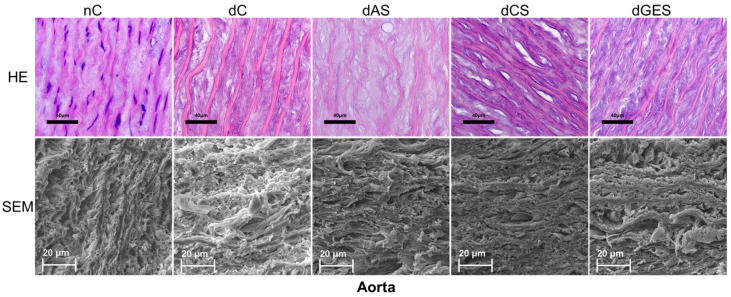
Samples of native and decellularized aorta (HE—hematoxylin-eosin staining, scale bar 40 μm; SEM—scanning electron microscopy, scale bar 20 μm).

Preservation of decellularized aortic samples in a CS and GES solution preserved the structure of the CTF to the maximum extent possible (uniformity of thickness of elastin fibers, degree of waviness of elastin fibers, structure of collagen fibers). SEM photographs of the same groups of materials show us similar results (Figure 7).

**Figure 8:**
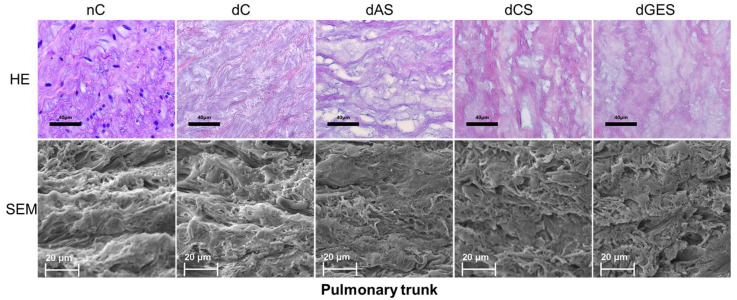
Samples of native and decellularized pulmonary trunk (HE—hematoxylin-eosin staining, scale bar 40 μm; SEM—scanning electron microscopy, scale bar 20 μm).

In native samples of the pulmonary trunk (nC group), elastin fibers were found to be predominantly circumferentially oriented, grouped in loose layers, with bundles of collagen and smooth muscle cells interspersed in the axial direction. After decellularization (dC group), the overall three-dimensional picture of the connective tissue matrix remained almost unchanged. During storage, it was shown that the antibiotic solution did not contribute to the preservation of the structure of the fibers; the degradation of the fibers occurred due to the partial dissolution of elastin and collagen with the appearance of voids, and the microfragmentation of elastin was observed. After storage in CS or GES solutions, elastin and collagen fibers are modified due to slight swelling and the presence of individual zones of elastin microfragmentation but maintain their sufficient waviness. In all groups except AS, the regularity of the structure of the connective tissue scaffold can be observed in scanning photographs (Figure 8).

## 4. Discussion

Currently, cryopreservation is one of the most common methods of long-term storage of biological tissues, as this method allows the functioning of the human tissue allograft bank to be ensured [29]. This method of preservation is not without its disadvantages, as it requires careful compliance with all stages of the freezing and thawing of the material, increased requirements for the technical reliability of the equipment, and possibly the search for new cryopreservation media [17,30].

As literature reviews show, there is still considerable scientific and practical interest in studying the characteristics and regularities of cardiac valve tissue preservation in a humid environment with additional tissue decellularization [31,32,33]. For example, some researchers have shown that the preservation of the mechanical and structural properties of decellularized tissue matrices is not affected even by prolonged wet storage in sterile physiological or nutrient solution for up to 12 months [20,34].

Glycerol solutions positively affect the quality of heart valve tissue, providing additional parameters for quality assessment during processing and storage. Wang et al. found that proteins present in decellularized heart valve scaffolds were stable during storage. The addition of glycerol was found to stabilize the proteins, which is a reversible effect that diminished after washing [35]. However, the pharmaceutical preservatives octanediol and phenoxyethanol have not yet found their place as biocidal agents for the non-cryogenic storage of decellularized heart valve extracellular matrix. In this work, for the first time, we have obtained results showing the fundamental possibility of using and further developing this technology.

The efficacy of decellularized heart valve transplantation largely depends on the components of the cell removal method (enzymatic, detergent, etc.) and the potential immune response after implantation [32,36]. The decellularization techniques and their variations are also used by other researchers, which proves the adequacy of this method in tissue engineering [33,37,38]. Previously, we published the steps in the development of our own protocol, where the effectiveness of detergent decellularization was determined by routing histomorphology, fluorescent staining for nuclei, immunohistochemistry for intracellular filamentous proteins, as well as the identification of the immunogenicity of the fabricated materials [11,39,40,41].

Strength is an important property of a deformable material, understood as the ability to resist destruction under the action of external forces. It is one of the main indicators characterizing the mechanical properties of the tissue, the quantitative assessment of which uses the value of the destructive stress (tensile stress) [37,42]. In the present study, decellularization of the AO material causes a significant increase in the value of tensile stress in the circumferential direction. At the same time, the value of Young’s modulus also increases, but without reliable differences from the values of the native control group. After long-term wet storage in alcoholic (CS and GEM) and non-alcoholic (AS) solutions, the main changes in mechanical properties were also evident in the tensile strength of the aortic wall material in the transverse direction. Almost all data on mechanical properties in longitudinal tension remained at the level of control values of the corresponding subgroups (decellularized and non-decellularized). Such anisotropy of biomechanical properties in the connective tissue scaffold of the aortic wall is explained by the peculiarities of the three-dimensional orientation of CTF fibers, which provides effective compensation of intravascular fluid pressure pulsation to maintain active blood flow [43,44].

The deformability of the material is reflected in the relative elongation under tension indicator. This indicator is influenced by the structure and fiber composition of the tissue [43]. Previously, it was shown that the main mass of elastin fibers in the aortic wall is located in the media as part of elastin lamellae, which are predominantly arranged in the circumferential direction [44,45,46]. Collagen fibers in the media are oriented at an angle of about 45° to the vessel axis, in the intima and adventitia, and more in the longitudinal direction. When determining the value of tensile strength, the maximum value of the applied force after which the specimen collapses is initially recorded. In fact, it depends on the number and quality of collagen fibers (mainly) present in the tissue. Apparently, a significant increase in the strength of aortic and especially pulmonary trunk specimens during storage in alcoholic solutions (CS and GEM groups) is probably related to a molecular change in the structure of the main CTF proteins, collagen and elastin. It is known that the three-helix structure of collagen, as well as the ratio of the hydrophilic and hydrophobic sites of protein molecules, changes under the influence of alcohols [46,47,48]. Our study has shown that the relative elongation of the AO material under tension in the transverse direction remains at the level of the control values in all the experimental groups except for the GEM subgroup. In this case, the elongation index is 1.58 times higher than that of the non-decellularized analogue, nGEM. A similar observation was made for native and decellularized pulmonary trunk samples. Decellularization resulted in an increase in both the longitudinal and transverse elongation of PT materials. In this situation, we should assume the effect of glycerol as a plasticizer on the structural organization of the connective tissue framework fibers of the aortic wall and pulmonary trunk. Some researchers have noted these changes in the biomechanics of materials stored in glycerol solutions [49,50,51].

Young’s modulus characterizes the stiffness of the material [37]. Irrespective of the direction of stretching, the values of this index in all experimental groups after storage in the studied solutions do not significantly differ from the indices of native tissues. Our comparison of Young’s modulus values under circumferential and longitudinal stretching within groups with the same type of tissue treatment shows significant differences in average values. Note the slight decrease in Young’s modulus values for decellularized pulmonary trunk samples in the transverse direction. The topography of the interposition of elastic fibers of the extracellular matrix can be used to explain this fact [44,50]. The decellularization of pulmonary trunk tissues decreases tensile strength and increases extensibility. The strength of PT material in the region of elastic deformation described by Young’s modulus is also slightly reduced, but this is compensated by the influence of the components of CS and GEM solutions. Further studies are needed to confirm this assumption. In total, the mechanical properties of decellularized materials of both aortic and pulmonary trunks do not change significantly after long-term wet storage compared to native materials. The described wet storage protocol using alcoholic solutions of glycerol or an octanediol/phenoxyethanol mixture may be intended for the further fabrication of extracellular matrix for tissue-engineered biological heart valve prostheses.

## Figures and Tables

**Table 1:** List of experimental groups.

**Group**	**Description**	**Subgroups**
C	Control	nC—native control samples
		dC—decellularized control samples
AS	Antibiotic solution	nAS—native samples in AS
		dAS—decellularized samples in AS
CS	Complex solution	nCS—native samples in CS
		dCS—decellularized samples in CS
GES	Glycerol-ethanol solution	nGEM—native samples in GEM
		dGEM—decellularized samples in GEM

## Data Availability

Data can be provided on request.
